# Slow Well-Being Gardening: Creating a Sensor Network for Radiation Therapy Patients via Horticultural Therapeutic Activity

**DOI:** 10.3390/s24123771

**Published:** 2024-06-10

**Authors:** Teng-Wen Chang, Shih-Ting Tsai, Hsin-Yi Huang, Yi-Sin Wu, Ching-Chih Chang, Sambit Datta

**Affiliations:** 1 School of Design, National Yunlin University of Science and Technology, Douliou 64002, Taiwan; ting72919@gmail.com (S.-T.T.); d10730018@yuntech.edu.tw (H.-Y.H.); wu.rilla918@gmail.com (Y.-S.W.); jingji.ued@gmail.com (C.-C.C.); 2 School of Electrical Engineering, Computing and Mathematical Sciences, Curtin University, Bentley 6102, Australia; sambit.datta@curtin.edu.au

**Keywords:** Internet of Healthcare Things, wearable sensor, life quality improvement, effective computing, emotional support

## Abstract

Well-being can reflect people’s psychological conditions and be used alongside physiological parameters to evaluate patients’ physical and mental health. The modern medical environment increasingly incorporates digital carriers, human–computer interaction devices, sensible spaces, and the execution of suitable algorithms. Slow design in healthy human–computer interaction is often used to reflect people’s dependence on or support from behaviors or objects, promoting the stability of behaviors as well as meaningful and positive changes. Therefore, in this study, we propose a slow sensing model, develop a Slow Well-Being Gardening system, and use it to evaluate behavioral data from radiation therapy patients during treatment sessions and horticultural therapy. This study is based on SENS and slow design, setting the hospital lounge as a sensible space and establishing a sensor system. After a 10-day inspection, the process was evaluated and verified. Ultimately, data from facial detection (smile) and HRV showed that the patients in the experimental group experienced a significant improvement in their well-being, feeling better than those in the control group who maintained the most common state in normal treatment. Therefore, it can be inferred that the Slow Well-Being Gardening model is indeed valid and can be further developed.

## 1. Introduction

Using health as the starting point, we aim to enhance patient well-being. This research approaches the topic from the perspective of design computing, with a focus on its development in the medical field, particularly in clinical radiation therapy applications. The modern medical care environment benefits increasingly from the intervention and assistance of digital carriers, human–computer interaction equipment, and sensible devices (or spaces). These technologies are designed to support the physical and mental health of users and patients [1]. This article will focus on the following exploration points: (1) Concentrate on design computing in the medical field, emphasizing clinical radiation therapy applications. (2) Employ digital carriers, human–computer interaction equipment, and sensible spaces to enhance patient well-being. (3) Develop interventions aimed at improving both the physical and mental health outcomes for patients.

### 1.1. Background

In modern research on “Well-Being”, the clearest definition of well-being is currently provided by the WHO: a state of happiness does not simply mean the absence of illness or weakness but encompasses being well physically, mentally, and socially. Being in a good state, “well-being”, in psychology, refers to emotional happiness and satisfaction [2]. In addition, psychological well-being, as defined in the literature, is characterized by feelings of happiness, contentment with life, and the absence of depressive symptoms [3]. Marjaana Sianoja et al. [4] pointed out that walking in an open space or park during breaks, as well as engaging in some relaxing activities, including sports, fitness, massage, and gardening, can improve concentration at work and reduce fatigue after work. These activities reduce feelings of fatigue and psychological discomfort, thereby enhancing well-being at work. Dopamine, also known as the “feel-good hormone”, is responsible for promoting “pleasure-seeking” behavior and driving the brain’s reward system, allowing humans to “set goals” and then “achieve tasks” [5,6,7]. If we feel a sense of accomplishment or are praised for good performance, or if we persist in executing a prepared plan, the brain will secrete “delicious dopamine”, making people feel pleasure and happiness [5]. In addition, happiness can lead to smiling, and smiling can also create happiness. Smiling triggers the release of serotonin and dopamine in the brain—chemicals that help people feel refreshed and happy. Smiling has many positive effects on the body, including lowering heart rate and blood pressure, reducing stress, and improving mental health [6,8].

With the rapid advancement of medical technology and its applications, treatment methods have also progressed quite rapidly. Although modern medical treatments have not completely eradicated cancer, many malignant tumors that were considered incurable in the past are no longer incurable [9]. Even in some cases, cancer can be managed like other chronic diseases such as diabetes and high blood pressure, and is not immediately life-threatening; conversely, patients can coexist peacefully with it.

Radiation therapy (RT), as a common and widely used treatment modality, plays a key role in cancer treatment. However, radiation therapy can also cause a series of physical and mental stressors for patients, including depression and other psychological disorders. British research found that only one-third of cancer patients experiencing psychological or emotional distress are willing to accept referrals [10], which also showed that female patients and those with moderate to severe depression were more likely to receive referrals [11]. Depression and anxiety symptoms are more common in breast and prostate cancer patients in outpatient clinics, while hospitalized and terminal cancer patients exhibit higher proportions of depression and anxiety states [12]. The psychology of patients fluctuates with the different treatment conditions of the disease. It is challenging for medical personnel to comprehend and assemble this type of information, making it difficult to provide appropriate advice to patients in real time.

With the improvement of treatment outcomes and longer survival periods, the psychological issues related to cancer treatment are increasingly valued by society. Horticultural therapy, a therapeutic approach based on gardening activities, is commonly used in various clinical settings to support physical and mental health [13]. Engaging in the process of planting and observing plants allows patients to feel the beauty and tranquility of nature and derive pleasure from it. Additionally, gardening activities provide an outlet for emotional expression and release, helping patients better manage stress and emotional distress [14]. Horticultural therapy can help improve mental health; studies have shown that participating in gardening activities can reduce stress, anxiety, and depression symptoms, as well as improve psychological well-being.

Slow design is a design approach that emphasizes the consideration of human needs and feelings in the design of spaces and environments. Slow design [15] based on slow technology, is often used to reflect people’s dependence on behaviors or objects and promote positive changes in stable behaviors and the meanings behind them. Additionally, slow design is used to understand people’s engagement, emotion, and intention in interactions. Therefore, a slow sensor for collecting slow activity information is a key element in slow design. In the radiation therapy environment, slow design can be used to create a comfortable and safe space or situation [16], which can reduce the psychological stress and anxiety of patients during treatment. Subsequently, by establishing suitable behaviors for patients undergoing radiation therapy, designers provide behavioral “goal setting“ and “achieving tasks”, allowing patients/users to experience a sense of well-being during the behavior process.

### 1.2. Motivation and Approach

At present—for both ordinary people and patients—emotions and well-being remain at a relatively intellectual or subjective stage. Despite the basic definitions provided by the World Health Organization (WHO), there is still no complete scientific model, and a truly objective viewpoint has yet to be quantified.

This article processes clinical physiological signals, which are commonly used by researchers to detect emotion-related clinical physiological signals. It aligns these signals with the patient’s behavioral performance in the situation [17,18,19], and utilizes algorithms suitable for recording long-term behavioral signals. It then introduces an implementation suitable for patients. Based on these verifiable, objective, and detectable behavioral tasks, which can be cross-analyzed by algorithms, a set of objectives, as well as clinical and scientific models, can be established.

To summarize the above descriptions, this study aims to enhance the process of radiation therapy by understanding the patient’s psychological state and behavior patterns, thereby improving the patient’s treatment experience and support effects. It focuses on patient-oriented psychological well-being [1,12], utilizing slow design to define behaviors and build a sense of well-being experience [13,14]. Therefore, this paper proposes a slow-speed sensor model based on the concept of behavior setting, which is specifically designed for the gardening activities of radiation therapy patients.

## 2. “Slow Well-Being Gardening” Framework

This research is based on the SENS smart space sensing network and the slow-design computing model (From: SOFT Lab, Douliou, Yunlin, Taiwan). It integrates conditions commonly used to evaluate well-being in recent years, including heart rate, heart rate variability, and facial expressions (smiling). The model “Slow Well-Being Gardening” integrates and measures well-being.

### 2.1. Sensible Space with IoT

Smart spaces focus on developing appropriate cooperation between users, spaces, and devices to achieve the best possible interaction and experience. Therefore, the goal of a smart space is to enable users to respond to interactions and changes between users and space through sensors that support the Internet of Things [20,21,22].

SENS [23] is a system implemented through smart environment equipment and new experience designs. Generally, the construction of smart environments corresponds to users’ habits, but the information does not necessarily match each user’s interaction. SENS operates through the connection of objects in the Internet of Things architecture, where devices can communicate via agents. This setup allows for the transmission of information between devices, enhancing the interaction between users and the environment. It presents information on a digital interface, providing all information in the most understandable way for users. This method, based on smart home design, develops co-creation space design. Through innovative design methods and processes, the integration of smart environments and user behavior continues to advance, driven by the development of hardware components for IoT sensors, making SENS design feasible.

Sensible space is a rapidly developing technology [24], and it is gradually being extended to remote medical and nursing care. Nowadays, there is a wealth of related literature and cases that utilize human-computer interaction and usage sensing methods to facilitate effective communication between patients and medical personnel. This includes the integration of smart health technologies, big data applications for patient data, external and implantable sensors, professionals’ acceptance of health information technology, and the use of complementary and alternative medicine. In addition, other areas that have developed rapidly in recent years and are more relevant to this study include healthcare systems based on the Internet of Things, and newer applications of preventive medical interventions [25,26,27,28].

For example, Liu [29] established a remote transmission system that utilizes IoT technology to intelligently locate and track patients’ physiological information, analyze the results into medical records, and store them in the monitoring system to facilitate remote diagnosis and monitoring. By using various sensible devices and monitoring equipment, including the space where the patient is, which objects are used, and devices worn on the body, the physiological data of the patient can be collected and analyzed in real time. These data provide valuable insights into the patient’s status and behavior, help the medical team to more fully understand the patient’s needs and responses, and enable the provision of timely psychological support and assistance during treatment [29,30,31].

**Figure 1:**
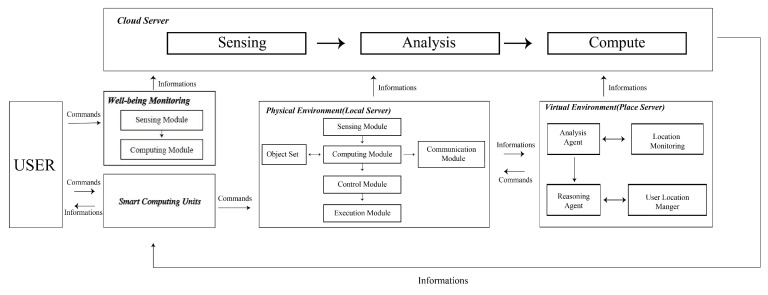
System flow of Slow Well-Being Gardening.

Sensible space technology has potential applications for radiation therapy patients, using sensible technology and data analysis to monitor patients’ daily activities, status, interactions, and other information [23]. In addition, this study also uses the smart space characteristics of SENS, combined with IoT and slow sensing systems, to construct a basic architecture for the operation of Slow Well-Being Gardening [23]. Through the application of Slow Well-Being Gardening, in addition to being able to observe the changes in the patient’s condition and depression, it can also integrate slow design and horticultural therapy to improve their quality of life and help improve treatment well-being (Figure 1).

### 2.2. Heart Rate Variability and Smile for Well-Being

Heart rate variability (HRV) and smiling, as measures of well-being, have attracted significant research interest in recent years. HRV is regarded as a physiological indicator that reflects the regulatory ability of the cardiac autonomic nervous system, and its variability is considered to be closely related to an individual’s psychological and physiological health status. Research shows that high HRV is often associated with better mental health and adaptability, while low HRV may be related to difficulties in coping with stress and regulating emotions. Moreover, the risk of cardiovascular disease in people with depression is 1.8 times that of people without depression [32,33,34,35,36]. In addition, the more days spent in a bad mood, the greater the likelihood of cardiovascular disease, with individuals who experienced poor mental health for 13 days being 1.5 times more likely to develop cardiovascular disease compared to those who reported no poor mental health in the past month [37].

At the same time, a smile, as a facial expression, is considered an important indicator of emotional and mental health. Past research has shown that smiling is associated with positive emotions and feelings of pleasure, and its expression not only reflects an individual’s current emotional state but may also have a positive impact on regulating emotions and social interactions. Smiling can trigger a true feeling of happiness in the brain, and research shows that the brain is connected to the immune system. Depression can weaken your immune system, and happiness, on the other hand, has been shown to strengthen the body’s resistance. Just smiling can have a huge impact on boosting your immunity. Even fake smiles are thought to reasonably reduce stress and lower one’s heart rate. Smiling helps reduce the body’s response to stress and lowers heart rate during stressful situations [8,38,39].

Integrating HRV and smiling—as assessment indicators of well-being—not only provides a multi-level assessment framework but also allows us to understand two different but interrelated factors: physiological and emotional indicators. In this study, HRV was measured through a wearable device, and Slow Well-Being Gardening was evaluated through sensors and facial expression recognition for smiles.

### 2.3. Horticultural Therapy (Horticultural Therapeutic Activity) on Patients

Nature, plants, sunlight, and fresh air are seen as essential elements of human life and are seen as healing forces that reduce work stress and ease the difficulties of daily life [40]. As a non-traditional treatment method, horticultural therapy has received more attention in recent years. Horticultural therapy promotes the physical and mental recovery of patients through the intervention of plants and the natural environment [12,41]. During the process of planting and observing plants, patients can feel the beauty and calmness of nature and gain pleasure from it. Clinically, horticultural therapy also has a positive impact on patients’ health. Gardening activities can increase patients’ exercise volume and promote muscle activity and joint flexibility. For example, growing flowers requires routine care, such as digging, irrigating, and harvesting, which can improve the patient’s physical strength and coordination, promote the functioning of the immune system, and improve the patient’s sleep quality. Horticultural therapy can also interact with oneself or other participants, which can help alleviate loneliness, and can also establish feelings of companionship and support [42,43,44,45].

In modern times, there have been more documents about the natural environment and plants, among which, pioneering studies on healing and sedative effects have been published; the classic ones are by Ulrich et al. [40,46], Marcus and Barnes [47,48], and Marcus and Francis [49]. In the highly cited work of Ulrich [50], the authors stated that even a natural scene outside a window can improve recovery after surgery [50].

Horticultural therapy has been shown to significantly impact various aspects of well-being, interaction, loneliness, and support. The literature covers the effects of horticultural therapy on stress reduction, cognitive function, mental health, and social well-being. The benefits for diverse groups such as the elderly, people with dementia, cancer patients, and those in long-term care facilities have also been highlighted. These benefits include improving mood, reducing loneliness, promoting social interaction, enhancing cognitive function, and providing a sense of purpose. At present, long-term treatment departments in most advanced countries have incorporated horticultural therapy into their treatment processes. Clinical departments in Taiwan have also accumulated related research in recent years, including in areas such as radiotherapy, chemotherapy, chronic disease treatment, surgical rehabilitation, and hospice care, all of which have seen interventions and related effects from horticultural therapy.

### 2.4. Clinical Application of Slow Design

Slow techniques are often applied to promote well-being, which is related to the slow design activities that we expected in the research [15,51]. Smart home technologies based on the Internet of Things (IoT) connect everyday objects, which is the basis of connectivity in the slow sensor model. Plants equipped with IoT and sensor networks will be closely related to the lives of patients in research. Sensors placed on the plants send signals according to the activity time of the patient’s treatment sessions. The sensors expected to be used are miniature motion sensors, soil moisture sensors, and face image sensors [52,53].

The study envisions building a set of slow sensible prototypes. Based on a slow design, the course activities of the majority of radiotherapy patients are simulated in the following steps: (a) Establish an appropriate daily activity for radiotherapy patients: Observe the user’s activity behavior over a long period of time within a given slow design target. (b) Design sensible systems and scenarios, then collect data. (c) Construct data schema and perform patient activity [54,55].

The use of slow technology and sensor technology is increasingly diverse in fields beyond design, including medicine, psychology, and material science. Although the above-mentioned related studies cover a wide range of topics, most of them cannot directly address a single specific situation covering “slow technology”, “slow design”, “well-being”, and “sensors”. Therefore, this study considers the specific application of integrating these elements as well as situational development. Examples include the use of slow technologies in medical contexts, combinations of sensors, measurement of well-being, and the use of sensors in treatments. However, the final evaluation requires a set of cross-comparison data that combines slow design, sensors, and well-being to provide more targeted and specific results. This will help construct a preliminary model of Slow Well-Being Gardening.

### 2.5. Sensor Application

Flexible electronic sensors are new-generation devices designed for comfort and user orientation. These wearable electronics, which can directly contact the skin, are soft, foldable, bendable, and resistant to deformation, revolutionizing human life. They can be attached to the skin, integrated into clothing, and provide an almost ideal medium for customized and personalized healthcare that patients can operate themselves. These low-voltage flexible electronic sensors not only comply with safe voltage standards for human use but also enable non-invasive, continuous, in situ, real-time, and comfortable monitoring of critical biological signals, offering clinically relevant indicators for preventive healthcare and disease diagnosis [56,57,58,59].

### 2.6. Summary

Through the research team’s analysis of related studies, we can understand the conditions under which well-being can currently be evaluated. Therefore, in this study, we integrated the above-mentioned “Slow Well-Being Gardening” framework and related technologies to extract elements suitable for use in Slow Well-Being Gardening and to establish frameworks and models.

The sensible space setting conditions of this study were determined after expert interviews (physicians, medical personnel), focus group interviews (patients), and field visits. These groups established a dedicated garden space for horticultural therapy in the hospital, allowing patients to participate in experiments. For the planting aspect of horticultural therapy, plant species suggestions will be provided during the research process according to the space’s suitability for light or special environments, with a focus on plants that are convenient for patients and horticultural activities. If the slow well-being design sensor model of this research can be successfully established and defined, in the future, the location and sensible method, then the size and type of planting can be selected according to the situation and field, and even the individual needs of patients. Research data are accumulated through slow design experiments: develop a set of gardening activities for radiation therapy patients, record the expression changes during gardening activities, then establish a system flow for slow sensor design to complete the accumulation of long-term treatment data, to improve the model proposed in this research.

In addition, this article specifically concentrates on advancing sensor architecture and algorithms capable of objectively detecting data, aiming to mitigate subjective influences in the well-being calculation process. Scholars in emotion and affective computing research currently employ expression detection and HRV independently; however, these two types of data have not been jointly analyzed to construct a comprehensive computing model, despite extensive literature support. Both expression detection and HRV are considered the most intuitive and objective physiological signals, forming the foundation for establishing a well-being algorithm in this study.

## 3. “Slow Well-Being Gardening” Research Design

Corresponding to the framework in Section 2, this chapter explains the process of constructing “Slow Well-Being Gardening” and includes evaluation methods and steps. According to the survey from focus group interviews, gardening is an activity that most cancer patients accept very well, and it appeals to a relatively wide age group. Through the settings of smart spaces and sensing systems, patients are provided with an ideal smart environment for interaction. Ultimately, the slow design purpose of this study was to provide a routine behavioral activity that would increase the companionship and well-being experienced by radiation therapy patients during their long-term treatment.

### 3.1. “Slow Well-Being Gardening” Smart Environment

**Figure 2:**
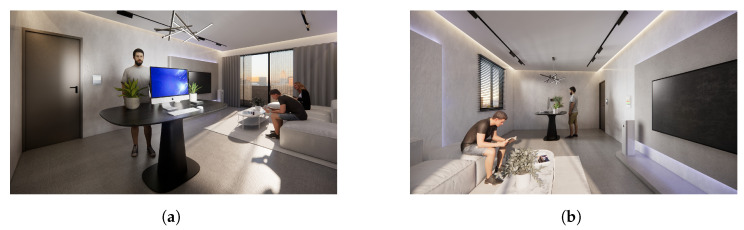
The lounge space for patients. (**a**) Usage situation of the experimental group. (**b**) Usage situation of the control group.

Location: The space for this study was set up at the medical center in New Taipei City, Taiwan, in collaboration with the Department of Radiation Oncology. The Department of Radiation Oncology provides a dedicated lounge for cancer treatment patients, which is well-lit, ventilated, and spacious. The purpose of this lounge is to allow patients to have a comfortable space to relax and wait for treatment before undergoing radiotherapy; they can also take a break and relax after the day’s treatment is completed. This space is also equipped with a TV and stereo, which can be used by patients if they need it (Figure 2).

Operation of Sensible Space: The sensible space designed and constructed by Slow Well-Being Gardening will need to record continuous events and information and be used to analyze the characteristics of information and situations, as well as potential events. These events and information will be considered by users. and applied in interactive situations. The algorithm will record the actions, positions, and behaviors that need to be observed in this study according to the timeline and will target the set tasks and analyze the activities in the space. Therefore, appropriate algorithms need to be used in the research. Through the establishment and analysis of the Slow Well-Being Gardening framework, we adopted the algorithm concept of slow design, which includes sensors, dynamic objects, static objects, and spaces. Subsequently, through human–machine interfaces, agents, and IoT, the system senses and records environments, people, conditions, behaviors, situations, and contextual information. When a user (patient) enters the lounge, which is a smart space, the cloud server begins to track the patient’s behavior and record their data. Additionally, the patient’s behavior triggers the sensing system to activate a Slow Well-Being Gardening scenario. These data and patient behaviors are continuously transmitted to the smart space server for integration, calculation, and analysis, and are stored on the cloud server.

**Figure 3:**
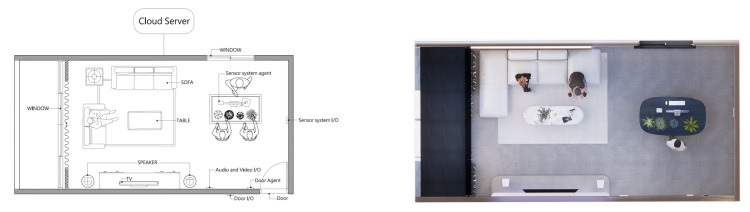
Smart space with agent—(**Left**): Distribution of agents in the smart space; (**Right**): Corresponding distribution of agents in the actual environment.

Agent Design: In the smart lounge space in this study (Figure 3), the agent design follows the communication framework in SENS, and the agent design includes (a) the sensor network and (b) behavioral data. The agents of the entire space include human users, lounge (control door, local server), lighting (ceiling lights and plant cabinet lights, local server), audio and video equipment (TV and audio, local server), and the local server. The gardening sensing system mainly explores and collects data in the research. This study is based on the agent framework of SENS and develops the path of Slow Well-Being Gardening. Between the various agents of Slow Well-Being Gardening, the sequence of communication events is as follows:

1. When the user enters the lounge, the manual agent is activated, notifies the agent coordinator, sends a signal to the lounge agent, and then confirms the user’s identity through communication with the cloud server.
2. After the reservation is established, the lounge agent notifies the door I/O to open and notifies and reserves each agent in the lounge through the agent directory.
3. The doorway and lighting system are activated first, and the agents of the audio-visual and plant sensing systems also switch from standby to power-on state. The cloud server provides initial instructions for each path and sends tasks to each ID.
4. If the user remains in the lounge and interacts with each agent after the cycle is completed, the agent will record data: whether audio/video equipment is used, whether the plant sensor is activated, and whether the facial recognition device is employed. It records whether these are started, the number of uses, and the duration of use. After recording, the data are sent back to the cloud server.
5. If an interruption signal is sent by a human, the lounge agent will be notified to confirm whether to continue or stop by checking the results.
6. When the entire process is completed, the data are sent back to the cloud server. Notify each agent in the lounge that I/O is closed.
7. The human agent interacts with the lounge agent through the interface program, and then logs out and leaves the lounge space.

### 3.2. Algorithms

Smart wearable device-HRV: To measure HRV, this study uses smart wearable devices, including smartwatches and smart bracelets from major brands. They are equipped with ECG functions and can measure Heart Rate and Heart Rate Variability. Finally, we will display ten days of Heart Rate Variability value.

Facial expression detection-Smile: The facial expression detection in this study is based on the interpretation mode of FaceReader, focusing on “smile” detection. We set the facial image sensor to record facial expression images and instructed it to capture a single image after detecting a smile lasting 2 to 3 s. During the patient’s expression process, as long as the calculation mode detects that the patient’s eyes, lips, and cheeks are at an angle close to a smile, and it lasts for more than 2 to 3 s, a complete smile will be detected. In this way, the number of smiles can be recorded while detecting. In addition to program interpretation, secondary interpretation testing by a psychosomatic physician (expert) is also available.

Algorithm Breakdown:

1. while detection() is True:

∘ This line initiates a loop until the detection() function returns. True. The detection() function is likely responsible for monitoring some activity (e.g., sensor data, motion detection) relevant to the user’s state.
∘ Without more context about detection(), it is difficult to. pinpoint the exact criteria for loop continuation. However, it is safe to assume the loop runs as long as the desired activity is detected.

2. hrv ← get_heart_rate():

∘ Inside the loop, the get_heart_rate() function is called, presumably to retrieve the user’s heart rate variability (HRV). HRV measures the variation in time intervals between heartbeats and can be indicative of stress levels, fitness, and overall health.
∘ The retrieved HRV value is stored in the variable hrv.

3. activity ← activity_analyze():

∘ The activity_analyze() function is called, likely to analyze sensor data or user input to determine the current activity. The function’s return value is stored in the variable activity.
∘ The specific implementation of activity_analyze() might involve machine learning models, pattern recognition, or rule-based systems to classify the activity (e.g., gardening, walking, resting).

4. if activity = gardening then:

∘ An if statement checks if the value of the activity is equal to “gardening”.
∘ If the condition is true (i.e., the user is determined to be gardening), the code within the if block executes.

5. record_heart_rate_var(hrv):

∘ This line calls the record_heart_rate_var() function, presumably to store the captured HRV value (hrv) for later analysis or visualization. The exact storage mechanism (e.g., database, file) depends on the program’s design.

6. expression ← emotion_analyze():

∘ The emotion_analyze() function is called, likely to analyze. sensor data or facial expressions (if camera access is granted) to determine the user’s emotional state. The function’s return value is stored in the variable expression.
∘ The specific implementation of emotion_analyze() might involve machine learning models for facial recognition and sentiment analysis.

7. if expression = smile then:

∘ An if statement checks if the value of the expression equals “smile”.
∘ If the condition is true (i.e., the user is detected to be smiling), the code within the if block executes.

8. smilecount += 1:

∘ If the user is smiling, a counter variable smilecount is incremented by 1. This variable keeps track of the consecutive number of times a smile is detected.

9. if the smilecount > 1 then record_smile(smilecount):

∘ Another if statement checks if the smilecount is greater than 1. This condition ensures that only sustained smiles (multiple consecutive detections) are recorded.
∘ if the smilecount is greater than 1 (i.e., a sustained smile is detected), the record_smile (smilecount) function is called. This function presumably stores the number of consecutive smiles (smilecount) for later analysis or visualization.

10. else smilecount ← 0:

∘ This else block executes if the user is not smiling or if a sustained smile has not been detected yet.
∘ In this case, the smilecount variable is reset to 0, effectively starting the smile detection process again.

Overall Purpose:

This algorithm is designed to monitor a user’s activity (focusing on gardening in this example) and capture their heart rate variability (HRV) during that activity. Additionally, it attempts to detect and record sustained smiles (potentially indicating enjoyment). The captured data (HRV, smile count) could be used for various purposes, such as the following:

- Understanding the relationship between gardening and stress levels: By analyzing HRV during gardening sessions, the system might provide insights into whether gardening is a relaxing activity for the user.
- Promoting positive emotions: Recording sustained smiles could be used to provide positive feedback or encourage the user.

The entire calculation process is as follows; Algorithm 1:   
**Algorithm 1:** Pseudocode of sensor system
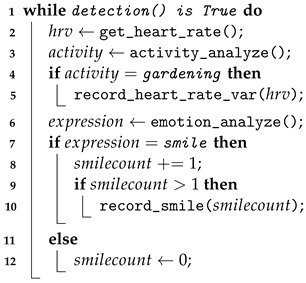



### 3.3. Processing Time

The duration of horticultural therapy varies depending on the case, objectives, and patient needs. Generally, in clinical practice, the duration can range from one week to several months [60,61], depending on the nature and goals of the therapy, as well as the individual circumstances and needs of the patients.

The choice of ten sessions (once daily) for our study was based on a synthesis of clinical case literature and expert interviews. The expert interviews included perspectives from (1) radiation oncologists, (2) respiratory therapists, (3) pulmonologists, (4) cardiologists, (5) radiographers, and (6) respiratory therapists, all of whom have deep clinical expertise in HRV. The interview dimensions also covered (1) establishing physiological signal data, (2) measurement duration, (3) sensing device configuration, (4) horticultural therapy integration, and (5) HRV frequency interpretation.

### 3.4. Sensor System of “Slow Well-Being Gardening”

**Figure 4:**
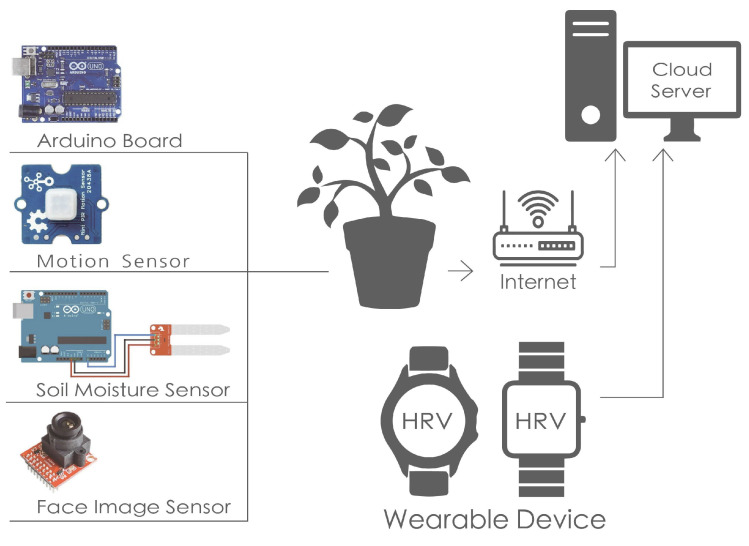
Sensor system of Slow Well-Being Gardening.

In the first step of the slow design sensor setup, horticultural planting (horticultural therapy) was identified as an activity suitable for the patient through expert interviews conducted in the study. As depicted in the construction diagram (Figure 4), the whole setup of the slow design sensor includes the following:

1. Motion sensor: grove—mini PIR motion sensor;
2. Soil moisture sensor: B12-5;
3. Face image sensor: Arduino OV2640;
4. Wearable device: smart watch—Apple watch.

The sensible system initiates its operation after the patient’s daily treatment session. Following the treatment, the patient has time to change clothes and rest. During this interval, the patient can proceed to the specially designed lounge, use the agent, and engage in gardening tasks such as watering or tending to plants, or participate in other activities during the break. When involved in gardening activities, the patient’s approach to the plant triggers the motion sensor, which detects the patient preparing to water or prune. Subsequently, the facial image sensor activates the camera to record the expression and time. Simultaneously, as the motion sensor detects the patient’s intention to water or prune, the measurement data are uploaded to the cloud server. Concurrently, the wearable device monitors HRV during gardening activities and links the acquired data to the cloud server. The sensing system incorporates a reminder feature. If the patient forgets to water the plant or neglects the gardening activities for an extended period, and the humidity sensor detects insufficient soil moisture, it triggers a smart assistant notification. This notification serves as a reminder for the patient to water the plant, ensuring its vitality and helping prevent the patient from feeling disheartened due to the plant wilting.

The primary reason for selecting the sensors used in this study was to provide a “modular sensor equipment” approach. Our goal was to enable the use of the simplest sensors in the most basic equipment environment. This sensor system includes physiological signal modules, behavioral signal modules, and environmental signal modules. Different clinical departments can match their required instruments accordingly, allowing for additions and interconnections. This not only increases the breadth of use but also enables direct replacement based on the needs of different departments.

### 3.5. Users: Patients (Unconventional Patient Conditions)

The subjects in this study are mainly patients receiving radiation therapy (all causes are cancer). Because cancer patients need to face longer-term treatment and emotional pressure, their participation in the test is not as enthusiastic as that of patients with common causes. The number of samples is also relatively small, which is unconventional compared to common disease patients.

Therefore, within the scope of this restriction, this study attempts to achieve homogeneity in the screening of subjects. Under the premise that all subjects are cancer patients, both the experimental group and the control group are consistently composed in terms of age range distribution (every 10 years as a step) and gender. Each group contains six subjects, making a total of 12 people in the two groups. The composition of the people is shown in Table 1, with the age range spanning from young to old.

Given that the study population comprises individuals undergoing long-term treatment, specifically patients receiving radiotherapy, the detection time is set at once a day for 15 min. The number of detections aligns with the patient’s regular treatment schedule over 10 days (or longer, depending on the type of cancer), allowing for the detection and calculation of preliminary results, an objective predefined by this study.

The subjects of this study were long-term clinical treatment patients with depressive tendencies. Therefore, data collection needed to be comprehensive, with careful consideration of time and face-to-face interactions.

This study was submitted for ethical review, which included a recruitment notice and informed consent form for participants. These documents outlined the study’s purpose, methodology, duration, and location. Additionally, they provided detailed explanations regarding participant privacy and data usage rights as follows:

(a) Before the test, experiment personnel provide explanations, and medical personnel offer instructions.

(b) Once the test begins, the patient conducts it alone, without interference from either the experiment or medical personnel.

(c) Data usage: Only the experiment staff has access to the facial detection data, which are securely stored on a dedicated hard drive. The data are destroyed after two years.

(d) Participants’ personal information and images are not disclosed or leaked. If participants consent to the use of their data for academic and research publications, they could indicate their agreement by opting in.

### 3.6. Evaluation Process

In terms of the evaluation process, patients undergoing radiation therapy will be asked to consent to participate in the experiment one day before their treatment, and then the researchers provide site options and planting types. After the selection is confirmed, the patient will be informed about the purpose of placing the plant and installing the sensing device. After the patient starts the course of treatment, he can go to the venue provided by the hospital for gardening activity after the daily treatment is completed. The miniature infrared motion sensor primarily functions as the system’s activation switch. When it detects that the patient is facing the plant and has raised their hand, the face recording sensor is triggered. Subsequently, the face recording and sensing system begins to document the patient’s facial expressions each time they engage in gardening activities. This facial recognition records the patient’s expressions when they are facing the potted plants and spending time before potting. Conclusively, if the patient has not watered the plants for more than two days, and the humidity sensor detects that the humidity is lower than 50%, the patient will be reminded to water through the IoT connection to prevent the plants from withering.

After the sensory accumulation data are collected, they will be transmitted to the Arduino by the entire set of sensors. The Arduino will then calculate the average value and upload the data to the cloud database. The gardening activity data collected over the ten-day course of treatment are accumulated using the aforementioned sensing paths and patterns. The subjects in the control group were not assigned tasks or instructions for gardening activities but were informed to use the lounge generously.

## 4. Evaluation Result

In the evaluation process of this research experiment, we used the prototype of the complete Slow Well-Being Gardening system on the subjects. In order to protect the privacy of patients, the photos were processed with mosaic and discoloration treatments (the photos are published with the patient’s consent).

### 4.1. Data Interpretation and Analysis

**Figure 5:**
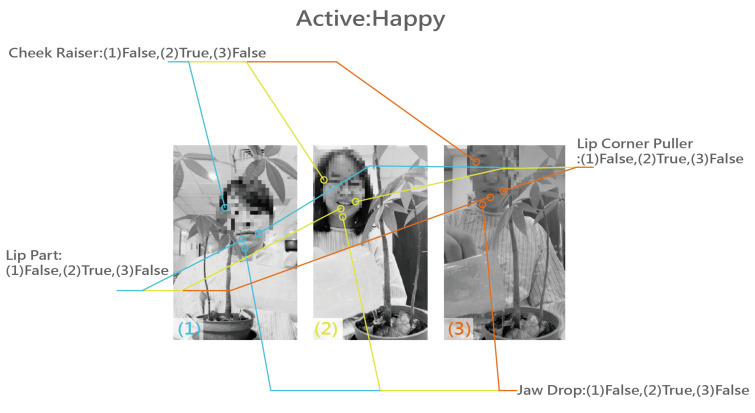
Facial expressions: smiling.

In terms of data analysis, this study primarily utilizes facial expression records and heart rate variability (HRV) data. Facial expression analysis focuses on quantifying smiles (Figure 5), while HRV is assessed as the average value (milliseconds, ms) during daily therapy and gardening activities. Expert recommendations from radiation oncologists, cardiologists, and specialists in physical and mental health suggest that HRV values may lack a fixed reference range due to various influencing factors such as physical condition, gender, age, immunity, and psychological status. In clinical practice, higher HRV values correlate with better heart rate sensitivity. Therefore, accurately interpreting this study involves using the patient’s first self-recorded HRV during the test as a baseline for subsequent comparisons.

In clinical HRV value interpretation, two primary determining factors are evident: (1) In ideal conditions, where both physiological and psychological aspects of human health are optimal, HRV values typically exceed 50 ms. However, due to numerous external factors influencing physiology and psychology, such as work, emotions, illness, stress, and sleep deprivation, values approaching or exceeding 50 ms indicate an improved heart rate sensitivity and are considered ideal. (2) In more common clinical scenarios, where HRV values may not consistently exceed 50 ms due to stress or various illnesses, interpretation relies on observing HRV trends over longer periods. If HRV can maintain stability or gradually increase over an extended timeframe, even if it does not reach the commonly accepted 50 ms threshold, it indicates an elevation in heart rate sensitivity and better overall health status.

**Figure 6:**
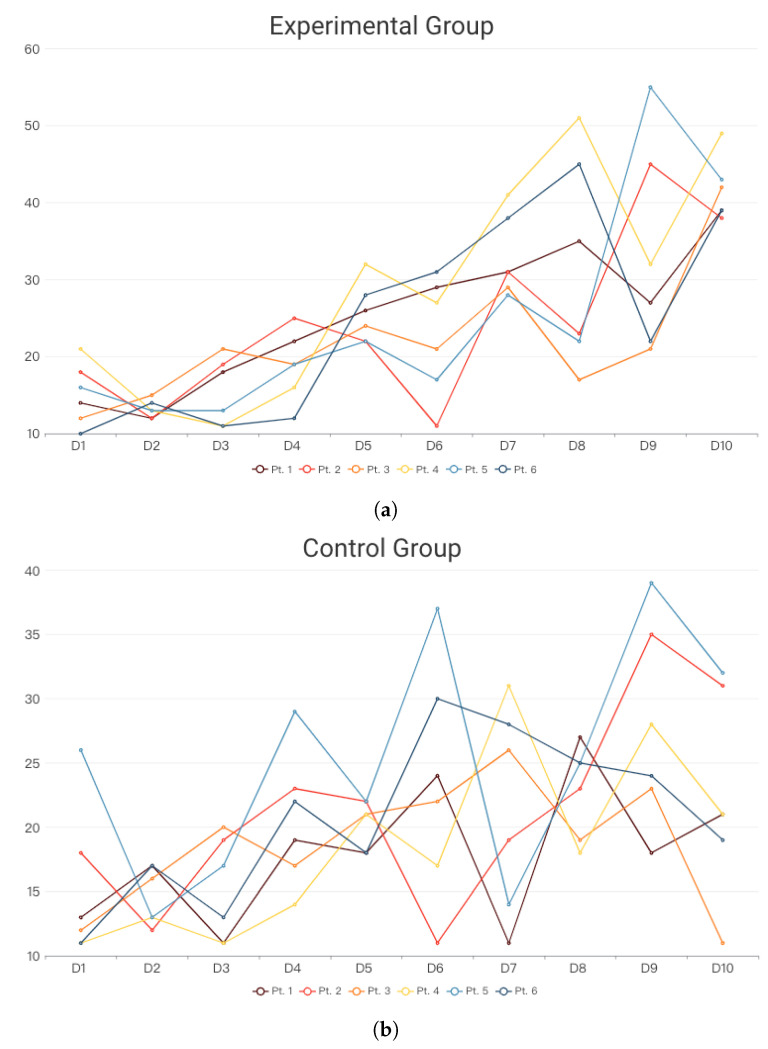
HRV data: x-days, y-HRV(ms). (**a**) Experimental group: The HRV curve displays a gradual and smooth increase. (**b**) Control group: the HRV curve exhibits irregular fluctuations.

The collected data are presented through information visualization line charts for the experimental group (Figure 6a) and the control group (Figure 6b), depicting HRV distribution over ten days for six subjects in each group (represented by lines of different colors).

**Figure 7:**
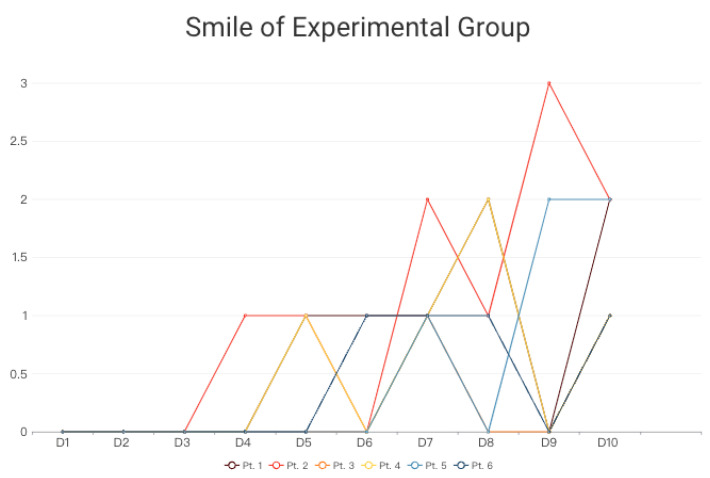
Facial expressions: smiling (x-days, y-facial expression counts).

Observations from the results indicate that during the initial day and week of radiotherapy, patients exhibit high tension, resulting in fewer smiles and lower HRV. However, around the third to sixth days, patients in the experimental group began displaying smiles, particularly in those undergoing the Slow Well-Being Gardening intervention (Figure 7). The visual data suggest that patients in the experimental group initially showed no expression but gradually exhibited smiles, with increased frequency in 2–3 patients. Most patients displayed a plateau in smile frequency closer to the end of the treatment and gardening activities, accompanied by an overall increase in HRV.

Additionally, a control group of patients undergoing conventional radiotherapy without horticultural therapy was organized. Due to the absence of intervention, smile detections in the control group were recorded as 0. Anxiety prevailed in the control group during treatment, resulting in predominantly low HRV within 10 days, with limited increases.

Comparing the two groups, the curve slope in the experimental group is relatively gentle, indicating less pronounced fluctuations and changes in HRV compared to the control group. Two patients in the experimental group showed HRV exceeding 50 ms, and the majority displayed an increasing trend compared to the initial measurement, signifying improved heart rate sensitivity due to horticultural therapy activities.

Conversely, the control group exhibited significant fluctuations in HRV values, suggesting unstable heart rate sensitivity. Although most subjects in the control group showed a gradual increase in HRV with treatment completion, nearly half did not exhibit a consistent rise similar to the experimental group. Moreover, the highest range of HRV values in the control group was only close to 40 ms, markedly different from the experimental group.

Based on these findings, we can infer that the Slow Well-Being Gardening model established in this study effectively alleviates patient anxiety and tension during treatment. This thoughtful model can be further developed and applied to foster well-being in other long-term therapeutic settings.

### 4.2. Second Layer Signal: Respiratory Rate

In the final study, the Bedside EKG clinical physiological sensor was utilized to detect the subjects’ “respiratory rate”, serving as the second layer of clinical physiological signal-assisted algorithm interpretation.

The primary purpose of the data was to demonstrate the analysis results through the essential link between HRV and respiratory rate. Slow, deep breathing can stimulate the autonomic nervous system, promote relaxation, and reduce stress, thereby increasing HRV. Therefore, in terms of the detection frequency range of the signal source, this study used the daily minimum average respiratory rate of six subjects in each experimental and control group as the comparison values for the two groups.

**Figure 8:**
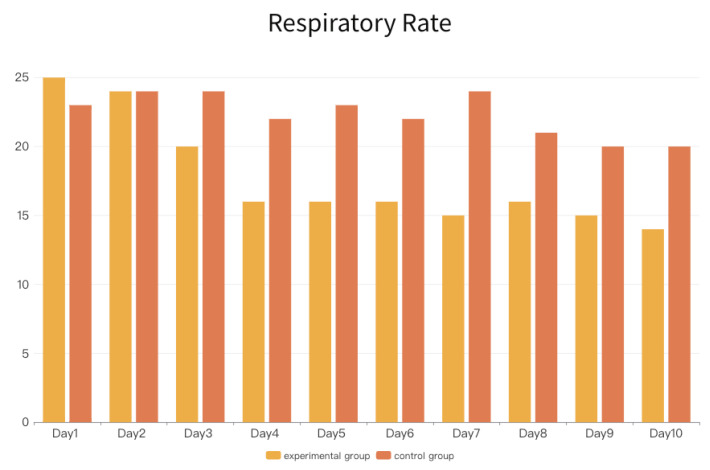
Respiratory rates for two groups (x-days, y-cpm).

Following the completion of radiation therapy on the first day, subjects in the experimental group began engaging in horticultural activities, while those in the control group remained seated indoors in a resting room (with the freedom to move indoors). Subsequently, from Figure 8, it can be observed that the average respiratory rate detected on the first day was 25 for the experimental group and 23 for the control group, with little difference between the two groups. Because both groups of subjects were undergoing radiation therapy for the first time, the data detected on the first day were generally more tense. Under normal circumstances, the normal respiratory rate for adults typically ranged from 12 to 20 breaths per minute, and it can be seen that both groups of subjects exceeded this normal range after averaging due to stress and pressure.

The data on the second day showed identical average values for both groups. At this point, it can be inferred that subjects in the experimental group, having undergone horticultural therapy, began to exhibit positive physiological signal development, while the control group showed a slight increase compared to the first day’s average.

From the third day until the end of the tenth day of the treatment, the experimental group showed a significant gradual decrease in the average respiratory rate, reaching the normal range for adult breathing. Although there was a slight increase on the eighth day, overall, a decreasing trend was observed, indicating a decrease in the respiratory rate and a gradual increase in HRV, which is consistent with the trend observed in Figure 6a for the experimental group’s HRV.

Moving on to the control group, the average respiratory rate showed slight fluctuations, with only the ninth and tenth days dropping within the normal adult respiratory rate range (less than 20 breaths per minute). The respiratory rate detection results of the control group, when compared with the HRV results of the control group (Figure 6b), correspond to the clinical trend of the relationship between HRV and respiratory rate.

### 4.3. Discussion

During the overall experiment, the research team observed unexpected interactions among the subjects. During the test on the 6th day, two patients entered the field before and after the incident, one of whom was a patient in the experimental group and the other was a patient in the control group. This experiment triggered an interaction, so they had a conversation, and the subsequent time they stayed in the field became longer. It was also recorded that the patients who talked had obvious changes in HRV. One patient was patient 1 (as seen in Figure 6a) and the other was patient 5 (as seen in Figure 6b). In particular, the HRV value of the control group patient was the most obvious, nevertheless, this is the only case. This study was conducted in a real medical field, so there would have been many actual emergencies. This interactive case has considerable reference value for future model establishment.

Through preliminary behavioral analysis of patients in this study, HRV and facial smile detection are currently available for cross-comparison. If future research raises the level of the algorithm, the recorded data can be summarized into more diversified interactive behavior classifications and algorithms, such as (1) sitting and resting, (2) listening to music, (3) watching TV, and (4) talking to people.

Regarding the number of participants, the subjects in this study are mainly patients receiving radiation therapy, all of whom are cancer patients. Because cancer patients face longer treatments and emotional pressure, their enthusiasm for participating in the study was lower when compared to patients with common illnesses. Consequently, the sample size is relatively small compared to studies involving patients with more common diseases. Initially, the number of participants was higher than the current six per group, but some patients unfortunately passed away due to worsening conditions, and others committed suicide due to severe depression. These are outcomes we deeply regret. Therefore, we hope this study can establish a foundational framework for emotion prediction, which will aid clinical applications by enabling medical staff to observe and intervene promptly.

In the final analysis of the research literature related to HRV, we discovered that most studies originate from various clinical disciplines. Therefore, the interpretation of HRV is frequently applied to the physiological values of that specific discipline, without a fixed range. It must be determined based on the circumstances of that particular medical field. Through the compilation and analysis of literature, as well as expert interviews with specialists, including cardiologists, pulmonologists, radiation oncologists, radiographers, and respiratory therapists, we concluded that the optimal interpretation of HRV depends on the patient’s physical and mental conditions and the environment they are in. Furthermore, a longer observation period is necessary to detect variations and differences. This conclusion aligns precisely with the situations and algorithms designed for patients in our study. Through the cross-analysis of clinical physiological values with contextual and behavioral factors, we aim to establish an objective algorithmic model.

## 5. Conclusions

Based on assessing and improving patients’ well-being, this research proposes a “Slow Well-Being Gardening” model. The Internet of Things technology combined with the network of slow happiness design sensors accumulates long-term data, which can help reveal the treatment behavior patterns and conditions of radiation therapy patients, and can also quickly adjust the goals in limited situations. In this study, the prototype of the slow well-being design sensor model was designed and developed for patients undergoing radiation therapy.

By implementing the Slow Well-Being Gardening process, we can initially explore the exact relationship between radiation therapy patients’ behavior and emotions during treatment. This study starts from the perspective of slow design, which can be developed in the medical field, and focuses on the application of clinical long-term treatment. Long-term treatment includes many types, such as radiation therapy, chemotherapy, targeted therapy, hypertension, heart disease, diabetes, and palliative treatment. It is believed that this model can be applied to these long-term treatments in more clinical fields. In forthcoming extended research, we will incorporate advanced detection models, e.g., machine learning and deep learning. By employing machine learning and deep learning, a more in-depth analysis of expression detection can be conducted. For instance, the angle of a smile correlates with the HRV frequency change. Through precise expression recognition using big data, it becomes feasible to discern whether it is a genuine smile. Future research can further explore the mechanisms of HRV and smiles—as well as their changes in different populations and situations—to more fully understand the application values of these indicators in well-being measurements, as well as consider more diverse environmental behaviors and complex human factors.

## Figures and Tables

**Table 1:** Experimental and Control groups.

Experimental Group		Control Group	
**Age**	**Gender**	**Age**	**Gender**
Subject (1): 26Y	biological female	Subject (1): 29Y	biological female
Subject (2): 37Y	biological female	Subject (2): 34Y	biological female
Subject (3): 44Y	biological female	Subject (3): 47Y	biological female
Subject (4): 54Y	biological male	Subject (4): 51Y	biological male
Subject (5): 56Y	biological female	Subject (5): 59Y	biological female
Subject (6): 62Y	biological male	Subject (6): 65Y	biological male

## Data Availability

Data are contained within the article.
